# The emotional intelligence of registered nurses commencing critical care nursing

**DOI:** 10.4102/curationis.v39i1.1606

**Published:** 2016-11-29

**Authors:** Yvette Nagel, Amanda Towell, Elzabe Nel, Fiona Foxall

**Affiliations:** 1 Department of Nursing Science, University of Johannesburg, South Africa; 2 School of Nursing and Midwifery, Edith Cowan University, Western Australia, Australia

## Abstract

**Background:**

Critical care is described as complex, detailed healthcare in a unique, technologically rich environment. Critical care nursing requires a strong knowledge base and exceptional clinical and technological skills to cope in this demanding environment. Many registered nurses (RNs) commencing work in these areas may lack resilience, and because of the stress of the critical care environment, coping mechanisms need to be developed. To prevent burnout and to enable critical care nurses to function holistically, emotional intelligence (EI) is essential in the development of such coping mechanisms.

**Objective:**

The aim of this study was to describe the EI of RNs commencing work in critical care units in a private hospital group in Gauteng, South Africa.

**Method:**

The design used for this study was a quantitative descriptive survey. The target population were RNs commencing work in critical care units. Data were collected from RNs using the Trait Emotional Intelligence Questionnaire – Short Form and analysed using the Statistical Package for the Social Sciences software.

**Results:**

The sample (*n* = 30) had a mean age of 32 years. Most of the participants (63%) qualified through the completion of a bridging course between 2010 and 2012. The majority (62%) of the sample had less than 2 years’ experience as RNs.

**Conclusion:**

The EI of RNs commencing work in a critical care environment was indicative of a higher range of Global EI, with the well-being factor scoring the highest, followed by the emotionality factor, then self-control, with the sociability factor scoring the lowest.

## Introduction

Nurses are considered to be the mainstay in the frontline provision of safe healthcare delivery (Buchan & Calman 2004); however, nursing shortages are a global phenomenon, leading to the current human resource crisis in healthcare (Beau 2006). Within South Africa, only 25% of registered nurses (RNs) working in critical care units are qualified as critical care nurses (De Beer, Brysiewicz & Bhengu 2011). Severe nursing shortages lead to the employment of newly graduated and inexperienced RNs in critical care units, often before they have developed appropriate professional knowledge and competence or are emotionally prepared for such a highly charged area of practice and the associated responsibility placed upon them (Ihlenfeld 2005; Oosthuizen & Ehlers 2007).

Lack of emotional preparedness can lead to stress and, ultimately, burnout, with nurses consequently leaving the critical care environment or indeed the profession. It is essential that nurses continue to be recruited to critical care units, but it is also imperative that experienced critical care nurses are retained, as their experience is invaluable in such a stressful environment (Mrayyan 2009). Kaur, Sambasivan and Kumar (2013) found that emotional intelligence (EI) can reduce the effects of burnout in nurses and improve their feelings of ownership towards their work. EI refers to the ability to identify, express, understand and regulate emotions, either negatively or positively, in oneself and in others (McQueen 2004).

Critical care nursing involves caring for patients who are suffering from life-threatening illnesses or injuries, while at the same time offering comfort and support to their family members (Rego *et al.* 2007). To care for such vulnerable patients and their significant others in a highly technological environment, nurses require a broad but specialist knowledge base and sound decision-making skills to function appropriately in such a stressful environment (De Beer *et al.* 2011). Thus, critical care nurses experience significant emotional labour as they manage their own emotions, as well as those of their patients and their significant others, adding further demands on caregivers (Stayt 2009).

RNs entering critical care for the first time may experience feelings of high anxiety regarding their performance, possibly related to the intensity of patient care; lack of knowledge; communication with unconscious patients; workload; role uncertainty; the feeling of being unsafe; making mistakes; new technology; and social acceptance (Li & Lambert 2008; Martins *et al.* 2009). Such anxiety may lead to extreme stress, leading to the decision to leave the critical care environment (Dodd-McCue *et al.* 2004).

A study by Shimizu, Couo and Merchan-Hamann (2011) found that the distress experienced by RNs was greater during their early career than those nearing the end of their career. The authors attributed this phenomenon to the pressure to learn to cope with the complex tasks needed to maintain the life of patients at risk of death; but once the skills were learned, they adapted to the distress. The stressful working environment may lead to younger nurses opting to leave the profession, as evidenced by the majority (74%) of the South Africa’s general RNs in 2009, over 40 years of age, with 23% between 30 and 40 years of age and only 3% younger than 30 years of age (Bateman 2009).

To prevent burnout and to enable critical care nurses to function holistically, EI is essential in the development of coping mechanisms (Patterson & Begley 2011). Codier, Muneno and Freitas (2011) support Akerjordet and Severinsson (2007) by suggesting that the strengthening of EI in nurses may impact on more positive attitudes, greater adaptability, improved relationships and increased orientation towards positive values, retention and prevention of burnout. Towell, Nel and Muller (2011) reported that mature and experienced critical care nurses appeared to have a higher range of EI. However, Scribante and Bhagwanjee (2008) found that almost half of all critical care nurses in South Africa were relatively new to the field of critical care nursing with less than 5 years’ experience.

The notion that intelligence is inherited, unchangeable and measured by traditional measures and standardised test scores is no longer universally accepted (Codier *et al.* 2011). Intelligence is now often described as a multifaceted concept that is developed throughout life. In 1993, Mayer and Salovey identified that individuals who ‘used emotions to facilitate their reasoning process’ functioned differently by not separating reason and emotion and were more effective in work, relationships and their own health and wellness (Mayer, Salovey & Caruso 2004, 2008). They coined the term ‘Emotional Intelligence’ and defined it as ‘the ability to monitor one’s own and others’ feelings, to discriminate among them, and to use this information to guide one’s thinking and action’ (Mayer, Salovey & Caruso 2004:159).

EI also involves possessing creativity, the ability to perform at an optimal level when completing tasks, hardiness and resilience, with ability to persist despite setbacks and failures (Bulmer-Smith, Profetto-McGrath & Cunnings 2009; Patterson & Begley 2011). Emotional skills are learned in childhood but can be developed and changed in later life with age, experience and through the influence of good role models and mentors (Begley 2006; Dulewicz & Higgs 2000; Goleman 1996). It is argued that an individual with higher than average EI is generally more successful in meeting environmental demands and pressures (Bar-On 1997).

Most EI theorists attempt to measure EI in accordance with the view that EI is an ability, a trait or a combination of both (Austin *et al.* 2005). The Trait Emotional Intelligence Model has been used as the theoretical framework for this study with the Trait Emotional Intelligence Questionnaire – Short Form (TEIQue-SF) used for data collection and analysis. Petrides, Pita and Kokkinakis (2007:284) explain Trait EI as ‘a constellation of emotional self-perceptions located at the lower levels of personality’, which refers to an individual’s self-perception of their emotional abilities, including behavioural dispositions and self-perceived abilities and is comprehensively measured by self-report and as such was suitable for this study.

The researchers consider that it is important to explore the EI of RNs commencing work in critical care units, leading to the question: what is the EI of RNs starting work in critical care units for the first time?

### Significance of the study

This study is important as it is assumed that if EI of RNs starting work in a critical care unit for the first time is known, then appropriate measures can be taken to address areas of low EI. This will assist RNs in the development of appropriate EI skills in order to better equip them to work in this challenging environment. The overall objective is to retain RNs in the critical care environment, as experienced RNs are invaluable to both hospitals and patients.

### Research purpose

The purpose of this study was to explore and describe the EI of RNs commencing work in critical care units.

### Research objectives

The objectives of the research were to describe the:

- Global Trait EI of RNs’ starting work in critical care units;
- well-being factor of Trait EI of RNs starting work in critical care units;
- emotionality factor of Trait EI of RNs starting work in critical care units;
- self-control factor of Trait EI of RNs starting work in critical care units;
- sociability factor of Trait EI of RNs starting work in critical care units.

## Research method and design

A non-experimental quantitative descriptive survey research design was used.

### Population and sampling

The study population comprised RNs starting work in critical care for the first time in one private healthcare group in Gauteng, South Africa. This research was exploratory and because of time and resource constraints in obtaining permission, only one private healthcare group was approached for the study. A benefit of using only one healthcare group was that all the respondents had similar group requirements to complete, for example, a clinical workbook.

From the 12 private hospitals belonging to the private healthcare group in Gauteng, 7 were selected, based on the number of critical care units and beds, as well as demographic accessibility. The hospitals chosen had two or more critical care units and more than 30 critical care beds. Convenience sampling was chosen as the hospitals were accessible and provided the means to conduct studies on a topic where probability sampling could not have been used to acquire information as suggested by Burns and Grove (2009). From the selected hospitals, five of the seven gave consent to conduct the research. The entire population who met the inclusion criteria were asked to voluntarily participate in the study. The inclusion criteria were RNs registered with the South African Nursing Council who had little post-graduate work experience in critical care, that is, had worked in a critical care unit for less than 3 months and were involved in direct patient care in the critical care unit.

### Data collection

The data collection instrument consisted of an informed consent sheet, biographical data sheet and the TEIQue-SF. The biographical data enabled the researcher to place the data from the TEIQue-SF into context regarding age, previous work experience as an RN, type of undergraduate education and the date of qualification.

### Research instrument

The TEIQue-SF is based on the Trait Emotional Intelligence Long Form version 1.50 (TEIQue). According to Petrides, Furnham and Mavrovelli (2007), the TEIQue is a scientific measurement instrument that is recommended for research as it provides a direct gateway to Trait EI theory. It aims to capture the affective aspects of personality as a whole. The TEIQue conceptualises EI as a personality trait and is composed of 153 items. The 153 items provide scores on 4 factors of broader relevance, 15 subscales as well as a Global Trait EI score. The items are responded to on a 7-point Likert scale, the scale ranging from 1 ‘completely disagree’ to 7 ‘completely agree’ (Pérez, Petrides & Furnham 2005). According to the Trait Emotional Intelligence Research Program, the scores reflect self-perceived abilities and behavioural dispositions and not cognitive abilities.

The 30-item TEIQue-SF was designed as an efficient measure of Global Trait EI and consists of 2 items from each of the 15 subscales of the TEIQue. The inclusion of items was based primarily on their correlations with the corresponding total subscale scores. This procedure was followed to ensure adequate internal consistencies and broad coverage of the sampling domain construct (Petrides 2011; Petrides & Furnham 2006). A priori factor score allows for the identification of four factors: well-being, self-control, emotionality and sociability (Petrides 2006). Permission was obtained from Dr K. Petrides to use the TEIQue-SF. It was selected as the most appropriate instrument for this concept based on a review of the literature and available instruments.

## Validity and reliability

### Validity

The TEIQue-SF is a standardised instrument and has measurement validity: it contributes to construct validity with models of differential psychology; it has discriminant and incremental validity vis-à-vis personality and has concurrent and predictive validity (Petrides *et al.* 2007). Theoretical validity was obtained from a content analysis of early models of EI, core elements that were common to more than a single model were included, while peripheral elements that appeared in only one conceptualisation were excluded (Petrides *et al.* 2007).

### Reliability

Reliability testing examines the amount of random error in the measurement technique and focuses on stability, equivalence and homogeneity, a reliability coefficient of 0.80 and higher is considered acceptable (Petrides 2006). Petrides reported a Cronbach’s alpha of 0.88 for the TEIQue-SF (Petrides 2006).

### Data collection process

Data were collected between September 2012 and July 2013. The researcher handed the data collection instrument to the clinical facilitators at the five hospitals for assistance with data collection. The clinical facilitators were identified as the main point of contact for new staff starting work in critical care and as such were in a perfect position to identify potential participants who met the inclusion criteria. The participants completed the consent form, biographical form and the TEIQue-SF and placed them into an envelope. The sealed envelope was then placed into a marked collection box by the participant, and the envelopes were collected by the researcher at various times throughout the study.

### Data analysis

A statistician was consulted to assist with data analysis, and SPSS version 19.0 for Windows was used. Descriptive statistics were calculated for biographical variables, mean total EQ and subscale scores. During data analysis of the Global Trait EI scores and priori factor analysis (as per the Trait TEIQue-SF scoring instrument) range, variance, mean and standard deviation were used to assist in interpreting the data.

## Ethical considerations

All participants signed a consent form, which included the research purpose and objectives, an explanation of the study and what the participants could expect through their involvement in the study. It also included details regarding assurance of anonymity, confidentiality and privacy, as well as a non-coercive disclaimer stating that their participation was voluntary and that if they refused to participate there would be no harm or negative consequences. The researcher’s contact details were included on the document should a participant have a query.

Ethical clearance was obtained from the ethics committee of the University of Johannesburg; the ethical clearance number is UNIV-2012–0023. An ethics clearance letter was obtained from the research committee of the private healthcare group in which the research was conducted.

## Results and discussion

The sample consisted of *N* = 30 RNs who started working in critical care for the first time between September 2012 and July 2013, in the five private hospitals selected for the study. Some of the participants did not answer all the items; therefore, the number of answers for each item is not always *N* = 30. This has been adjusted in the analysis.

### Biographical data

**FIGURE 1:**
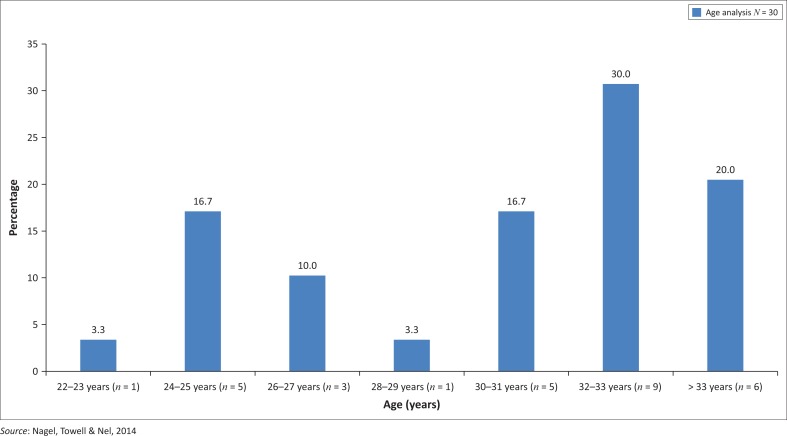
Age analysis of sample.

Figure 1 shows the analysis of the age of the sample. As the statistics obtained from the South African Nursing Council (2009) revealed that 61% of RNs in South Africa were between the ages of 40–59 years old, this indicated a younger sample size, as the mean age in this study was 32 years. In comparison with the South African Nursing Council statistics (2009), only 12.3% of RNs were under the age of 34.

The biographical data of the sample are shown in Table 1. The majority of the participants qualified through completion of a bridging course (from enrolled nurse to RN) (63%), between 2010 and 2012 (62%), with 76.8% of the participants working in wards in the hospital before starting in critical care. This points to an inexperienced sample of RNs, as 62% had less than 2 years’ experience.

**TABLE 1:** Biographical data of the sample (*N* = 30).

Variable	Qualifications	% (*N* = 30)
Basic qualification	Bridging diploma	63.3% (*n* = 19)
	4 year diploma	26.7% (*n* = 8)
	Professional degree	10% (*n* = 3)
Years since qualification	< 2 years’ experience as a registered nurse	62% (*n* = 18)
Previous work experience	Previous work experience in high dependency units but not in critical care units	13.3% (*n* = 4)
	Worked in wards in hospital prior to moving to CCU	76.8% (*n* = 23)
	No previous work experience, other than student experience	9.9% (*n* = 3)

Source: Nagel, Towell & Nel, 2014

With the severe shortage of nurses, it is important to know the background of the RNs starting in critical care to assist the transition into critical care nursing for newly recruited critical care nurses, thereby improving the retention of staff.

The Global EI score as an overall view of the EI of the sample will be presented first; thereafter, a detailed description of the four factors of EI of the sample will be presented.

### Global emotional intelligence

**FIGURE 2:**
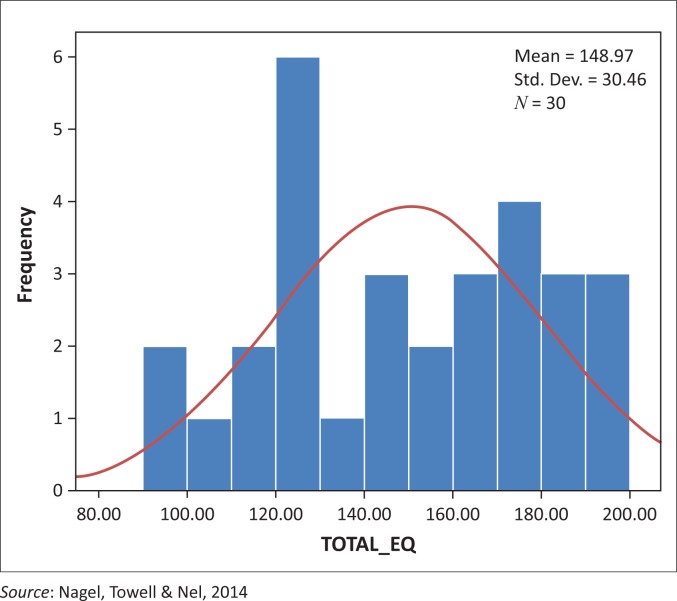
Global emotional intelligence scores of registered nurses starting in critical care (*N* = 30).

The data collected using the TEIQue-SF generated a Global EI score. Figure 2 displays the EI scores of the participants (*N* = 30) in the study. The histogram displays a unimodal, mesokurtic, slightly negatively skewed curve.

The possible range of the Global EI when using the TEIQue-SF questionnaire is 30–210 (it is a 7-point Likert scale ranging from 1 to 7, on 30 questions). The mean score was 148.9; the range in the study was 103, with the lowest EI score being 94 and the highest being 197. This, together with the variance of global 927.83 does indicate a wide dispersion of scores, indicating that some participants have a very low Global Trait EI, while other participants scored very high.

Towell *et al.* (2011) report a mean Global Trait EI score of 155.98, which indicated a higher range of EI than this study. The sample in the study (*n* = 220) by Towell *et al.* (2011) was found to be mature, experienced RNs, with 70.9% being trained in critical care nursing. Although the biographical data from the study by Towell *et al.* (2011) differs tremendously from that of the sample in the study, the difference between the Global Trait EI was only 7.01 points. The mean of 148.97 in this study indicated the global EI to be in the higher range of EI. This can be viewed in conjunction with the biographical data of the participants who (76.8%, *n* = 23) had previous work experience in hospital wards as an enrolled nurse (EN) and this may have influenced their EI.

### Analysis per emotional intelligence factor

**FIGURE 3:**
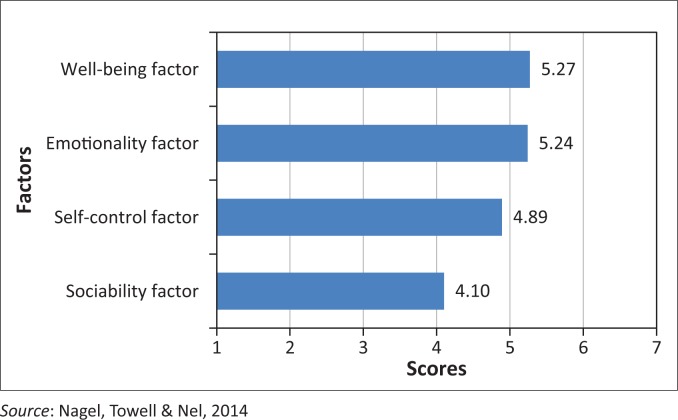
Scores on the four factors of trait emotional intelligence (*N* = 30).

The priori factor score allows for the identification of four factors: well-being, self-control, emotionality and sociability (Petrides 2006). The scores were derived from the 7-point Likert scale of the TEIQue-SF, with a minimum of 1 and maximum of 7; the values must be interpreted in terms of this scale. See Figure 3 for a summary of factor scores.

The participants perceived themselves to be strong, with a mean of 5.27 in the well-being factor. This factor consists of three facets: happiness, optimism and self-esteem. High scores in this factor reflect a generalised sense of well-being, extending from past achievements to future expectations. Overall, individuals with high scores feel positive, happy and fulfilled. In contrast, low scorers tend to demonstrate a low self-regard and respondents tend to be disappointed with their life. The well-being score largely depends on the scores on the other three factors of the TEIQue (Petrides 2011; Thomas International 2012).

The emotionality factor is the second strongest factor with a mean of 5.24 and consists of four facets: empathy, emotion perception, emotion expression and relationships. Petrides (2011) explains that individuals with a high score in the emotionality factor believe that they have a wide range of emotion-related skills. They can perceive and express emotions and use these abilities to develop and sustain close relationships with important others.

The third factor is self-control, scoring a mean of 4.89 and consists of three facets: emotion regulation, impulse control and stress management. The self-control factor describes how well the participants regulate external pressure, stress and impulses. According to Petrides (2011), high scorers demonstrate a healthy degree of control over respondents’ urges and desires. In addition to fending off impulses, respondents are good at regulating external pressures and stress. They are neither repressed nor overly expressive. In contrast, low scorers suggest that respondents are prone to impulsive behaviour and seem to be incapable of managing stress. Low self-control is associated with inflexibility (Petrides 2011).

The participants perceived themselves to be weakest in the sociability factor, with a mean of 4.1. This factor consists of emotion management, assertiveness and social awareness. The sociability factor differs from the emotionality factor in that it emphasises social relationships and social influence. It describes the ability to socialise, to manage and to communicate with others. The focus is on the individual as an agent in different social contexts rather than on personal relationships with family and close friends. Individuals with high scores on the sociability factor are better at social interaction, believing they have good listening skills and can communicate (Petrides 2011; Thomas International 2012).

The RNs in this study scored well on the EI questionnaire, with the well-being factor scoring the highest, followed by the global emotionality factor, then self-control, with the sociability factor scoring the lowest. This indicates that the sample see themselves as positive, happy and fulfilled individuals who are able to regulate external pressure and stress and are able to control impulses. The participants believe they have a wide range of emotion-related skills, are able to perceive and express emotions and are socially aware. The weakest scores were within the social context; participants did not perceive themselves as assertive.

## Discussion of results

RNs starting in critical care had a mean age 32 years, had mainly completed the bridging diploma from enrolled to registered nursing, had minimal previous work experience as an RN and most of their experience was within hospital wards. Participants completed the TEIQue-SF within the first 3 months of commencing work in the critical care unit. The EI of the participants was found to be in the higher range with the well-being factor scoring the highest, followed by self-control and then emotionality; sociability was found to be the weakest factor.

Freel (2009) found that RNs generally, have higher EI abilities than other occupations; furthermore, Budnik (2003) stated that nurses with a Masters degree have a higher EI compared to those with associate degrees. This indicates the importance of continuing education and training after completion of a basic nursing degree or diploma. Continuing education can also lead to personal enrichment; as Beauvais (2012) states, the skills associated with EI can be improved with education. Additionally, it benefits both the individual and the organisation as EI skills may help nurses establish and maintain a caring environment and cope with stressful work demands, leading to lower burnout and turnover of staff (Beauvais 2012; Kaur *et al.* 2013).

The facilitation and support process of RNs entering the critical care environment is of paramount importance in the quest to recruit and retain RNs in this specialist area of nursing, through support and guidance to ensure they develop and continue to enhance their roles as competent, effective and safe practitioners.

It is recommended that an assessment of the RNs’ Trait EI, including the 4 factors and 12 facets, should be undertaken as part of an orientation package within the hospital, to enable the unit manager, clinical facilitator or mentor to identify areas in which the RN might benefit from support and guidance. In doing so, the time and funds spent by the hospital on training and orientation could have an improved investment return in terms of retention of staff.

## Limitations

Simple random sampling would have been the preferred method of sampling in a quantitative study. However, as the researcher was based outside of South Africa during the study, there was no direct access to the new RNs, which could have led to lower recruitment. Additionally, the researcher was not able to identify potential participants, adding to the difficulties in recruitment. Because of geographical location, the study was conducted in one area of South Africa, with the use of one private healthcare sector. Therefore, the results are limited to the five hospitals belonging in the Gauteng province and cannot be generalised.

The amount of practical experience as an EN of the 63% (*n* = 19) of the sample (*n* = 30) who had completed a bridging diploma is not known, but this experience would be beneficial to the RN.

## Recommendations

The importance of continuing education after the completion of a basic nursing degree or diploma can lead to personal enrichment and enhanced EI (Beauvais 2012). This benefits both the individual and the organisation as well-developed EI skills may help nurses establish and maintain a caring environment (Beauvais 2012). This in turn, helps nurses to cope with the stressful demands of their work, leading to lower burnout and staff turnover rates (Beauvais 2012). The following recommendations for continuing education to facilitate EI are proposed based on the findings of this study:

Every critical care unit should employ experienced, expert nurses, who have a highly developed EI enabling them to act as mentors, encouraging EI growth in RNs entering the critical care environment.

Education relating to EI, specifically relating to the 4 factors and 12 facets of Trait EI should be undertaken in critical care areas. Nooryan *et al.* (2011) investigated the effect of teaching EI items to nurses working in critical care areas by implementing training programmes emphasising the recognition of emotions and their effects on internal and external aspects of the person, as well as relaxation and concentration exercises. The result demonstrated a positive relationship between high EI and low levels of stress and anxiety.

## Conclusion

The RNs in this study were found to be mature, had mainly completed the bridging diploma from an EN to RN, and had minimal previous work experience as an RN and the majority of their experience as ENs was in hospital wards. The EI of the participants was found to be in the higher range, with the well-being factor scoring the highest, followed by self-control and then emotionality, with sociability being the weakest factor. In closing, the RN is emotionally involved in the act of nursing, but there is a requirement to be educated regarding the need to nurture and care not only for their patients but also for themselves (Budnik 2003).
